# A Survey of Community Perceptions on Brain Donation for Research

**DOI:** 10.1089/bio.2023.0158

**Published:** 2025-02-13

**Authors:** Alicia Sweeney, Amanda Rush, Julia Stevens, Greg T. Sutherland

**Affiliations:** ^1^ New South Wales Brain Tissue Research Centre, Charles Perkins Centre and School of Medical Sciences, Faculty of Medicine and Health, The University of Sydney, Camperdown, Australia.; ^2^ Menzies Centre for Health Policy and Economics, Charles Perkins Centre and School of Public Health, Faculty of Medicine and Health, The University of Sydney, Camperdown, Australia.

**Keywords:** brain banking, postmortem, donor recruitment, perceptions, public

## Abstract

Postmortem brain donation for medical research is a little-known form of organ donation. While most brain research is carried out using animal models, many neurological diseases are uniquely human. Greater availability of human postmortem brain tissue from diseased individuals and controls would therefore improve the development of treatments for neurological and neuropsychiatric diseases. Globally, organ donation for medical research is dwarfed by organ donation for transplantation. In 2021, 36% of Australians were registered organ donors for transplantation, with public “in-principle” support even higher, at 76%. In contrast, there are little data on Australian or international brain donation rates for research. A 30-item online survey was conducted to ascertain knowledge of, and attitudes toward, brain donation in Australia. Of the respondents, 12/237 (5%) were current brain donors and excluded from further analysis. Of the remaining 225, 75% were registered organ donors for transplant. The vast majority (*n* = 189/225, 84%) of respondents supported or strongly supported the principle of brain donation. However, of those registered for transplantation or whole-body donors, 93/170 (55%) were not aware that brain donation was possible, while 50%, alternatively or also, thought that registering as an organ donor for transplantation rendered them a brain donor by default. Only 9/225 (4%) respondents indicated that they would definitely not donate their brain in the future, while 27 remained unsure. There is prominent public support for brain donation in Australia, with 84% of respondents willing to donate their brain. Yet, the extent of public misconceptions on brain donation for research suggests the need for further education on all types of organ donation, so individuals may make informed decisions.

## Introduction

Brain donation by healthy volunteers makes an important contribution to the understanding of normal brain function. Healthy brain tissue also serves a critical role as comparator in brain disease research. Yet, participation rates in brain donation research programs remain relatively low. It is not clear whether this is due to a lack of awareness of the programs, lack of being approached for participation, or specific reticence regarding donating brain tissue after death.

Postmortem organ donation is generally considered for three distinct purposes: transplantation to save or improve the quality of life of recipients; anatomy education and surgical training; and medical research. Of these, organ donation for transplantation is the most well known and widely practiced, with 76% of Australians indicating public support in 2021, although only 36% are registered to be a donor.^1^

Organ donation for transplantation has been managed at a national level through an online registry in Australia since 2009. While there has been some research on perceptions of whole-body donation programs for education and training purposes,^2,3^ there is a dearth of information on the public's perception of organ donation to support health and medical research in Australia.

Tissue and organ donation for research is facilitated by biobanks, with specialized biobanks focusing on postmortem brain donation. Recruitment of brain donors for research generally occurs through prospective donor programs, whereby a participant registers years before their death, providing medical and lifestyle information over their remaining life span. Prospective donor programs are most often associated with referral clinics for specific neurological diseases, where recruitment is facilitated by clinicians working directly with patients.

Separate brain bank models facilitate prospective recruitment of healthy donors through community-based programs or retrospective recruitment through the coronial system. The New South Wales Brain Tissue Resource Centre (BTRC) at The University of Sydney operates with both models, including a community-based prospective donor program, “Using our Brains,” to facilitate collection from healthy controls.^4^

A challenge for community-based brain biobanking is a low rate of brain donation. A persistent cross-cultural explanation for this is that potential donors consider the brain “special” or different from other organs.^5–7^ However, evidence also exists to the contrary, with a survey of 200 older British adults finding that just 5% of respondents considered the brain different enough from other organs for it to influence their decisions about donation.^8^

Another survey of 3279 community perceptions of brain donation in the United States identified that the major reason for nonparticipation was a lack of being approached.^9^ Furthermore, a survey of 1249 community residents in China showed that almost two-thirds of respondents were unaware that brain donation for research was possible.^10^

Based on these variable community perspectives on brain donation, and in the context of the known high level of support for organ donation for transplantation, we conducted a survey to understand perceptions of brain donation from a broad cross-section of the Australian community who were not currently enrolled to donate their brain for medical research.

## Methods

### Survey design

A 30-item online survey (Supplementary Data S1) was designed to ascertain community knowledge of, and attitudes toward, brain donation for medical research. Questions were developed by a team of experienced brain biobankers in consultation with current literature.^11^ The two-part survey was deployed in REDCap (Research Electronic Data Capture), a secure, web-based software platform hosted at The University of Sydney.

Part 1 comprised 11 demographic questions (8 drop-down responses, 1 yes/no question, and free text responses for age and postcode). Part 2 comprised 19 questions on attitudes and intentions regarding brain donation, summarized in Table 1. This included questions on organ donation for transplantation to differentiate this concept from postmortem brain donation for research.

**Table 1.** Overview of Survey Questions (Part 2)

Theme	Survey question topics
Perceptions and status of organ donation	Consideration of organ donation, comfort level for talking about organ donation, current organ donation status, registration approach, reasons for registration, and in-principle support for organ donation for transplantation
Support of others for organ donation	Confidence in family support and discussion of intent with others
Perceptions on brain donation	Reasons for registration; reasons for being an organ donor, but not a brain donor; reasons for not registering for brain donation; in-principle support for brain donation for research; and intent to donate brain
Support of others for brain donation	Confidence in family support and discussion of intent with others
Awareness of promotion	Recollection of advertisements for organ donation and recollection of advertisements for brain donation

Of the 19 questions in Part 2, there were 7 “select all that apply” questions on motivations and barriers to donation, 3 yes/no questions on donor status and knowledge of existing processes, 5 five-point Likert scales, and 3 drop-down select-1-response questions. Where relevant, survey respondents were invited to provide further explanation using free text boxes, with a final comment box option at the conclusion of the survey.

### Survey distribution and conduct

The survey was approved for distribution by The University of Sydney Human Research Ethics Committee (approval number 2020/423). It was open for participation for a 27-month period from July 2020 to September 2022. Survey respondents were recruited from a broad cross-section of the Australian community through Facebook advertisements and promotion in BTRC partner organization newsletters.

Participants were provided details about the background and context on the use of their responses, with consent implied by participation. Study data were collected and managed using REDCap. All survey questions were mandatory (respondents could not progress further unless an answer was provided). Exclusion criteria were residing outside of Australia, being aged under 18 years (the age of majority in Australia), and/or being currently registered with a brain donor program.

### Survey analysis

Following recruitment close-off, raw data from REDCap were downloaded, with descriptive statistics (mean, range), proportions, and associated figures generated in Microsoft Excel. Chi-square tests were employed to explore relationships between responses and demographics such as income and rurality (JMP, v 16.1.0; SAS Institute, Inc.). Free text responses for two questions (questions 18 and 30, Supplementary Data S1) were thematically analyzed by three independent coders for inter-rater reliability.

## Results

Two hundred thirty-seven respondents throughout Australia completed the online survey. Twelve respondents (5%) were already participants in a brain donor program. These respondents were excluded, leaving 225 respondents for further analysis aimed at exploring why there is not greater participation among Australians.

**FIG. 1.**
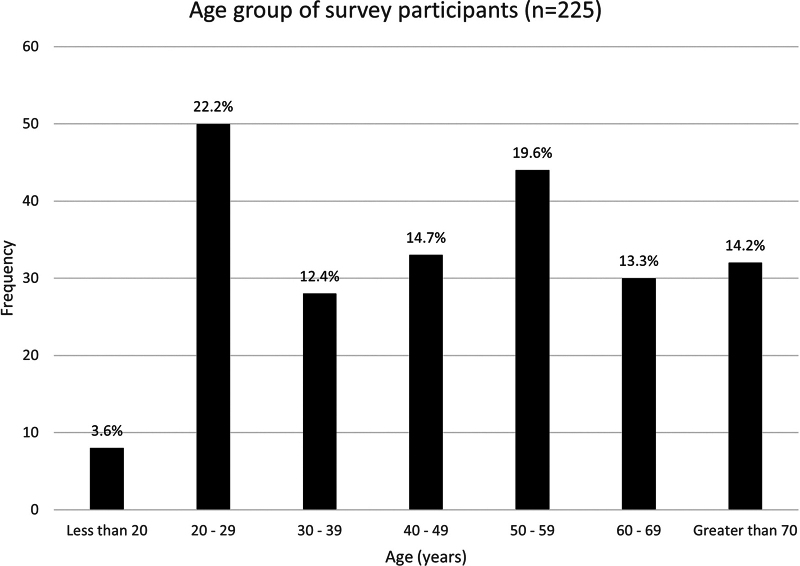
Survey respondents by age group. A histogram shows the frequency of participants in seven arbitrarily defined age groups (<20 years old, 20–29 years old, 30–39 years old, 40–49 years old, 50–59 years old, 60–69 years old, and over 70 years old, *n* = 225). Percentages are shown at the *top* of each bar.

Respondents were aged between 18 and 80 years (Fig. 1), with a mean age of 47 years. Most respondents were female (*n* = 169, 75%). Over half reported being single, divorced, widowed, or separated (*n* = 122, 54%), with the remaining 103 respondents (46%) either married or in a *de facto* relationship. One hundred ninety-seven respondents (88%) identified as White, reflecting the broader Australian population,^12^ with English being the home language for almost all (*n* = 218, 97%).

**FIG. 2.**
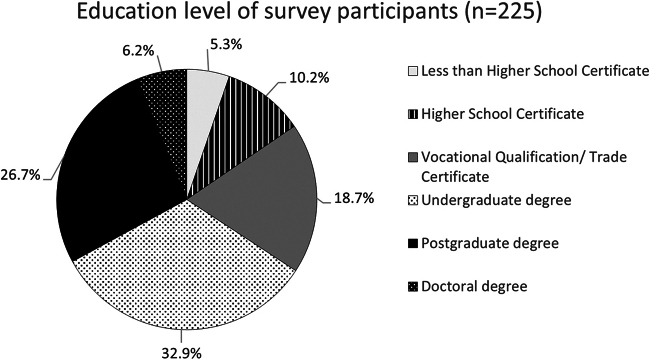
Level of education. A pie graph showing percentages (%) of educational achievement for all survey respondents (*n* = 225). Participants could select one of the following options: doctoral degree, postgraduate degree, undergraduate degree, vocational qualification or trade certificate, high school certificate (completed the final year of high school), or less than high school certificate (did not complete the final year of high school).

Approximately two-thirds of survey respondents had attended university, which was a higher proportion than for the overall Australian population^13^ (Fig. 2). Seventy participants (31%) self-identified as religious, slightly lower than the general Australian population.^14^

Eighty-three percent of respondents lived in an urban area, which is similar to the proportion of urban residents reported in the 2021 Australian census (86%).^15^ The 198 respondents who reported their income were stratified into two similarly sized groups who earned less than (*n* = 92) or equal to/more than $A90,000 per annum (*n* = 106).

Three-quarters (*n* = 168/225, 75%) of respondents self-identified as organ donors for transplant, of which 13 were also whole-body donors for medical education along with two others (*n* = 15). Forty-six respondents (20%) were either not registered for any form of organ donation or unsure of their donation status (*n* = 9/225, 4%). Thus, 170 respondents had registered to donate some or all their organs for transplant or teaching purposes.

**FIG. 3.**
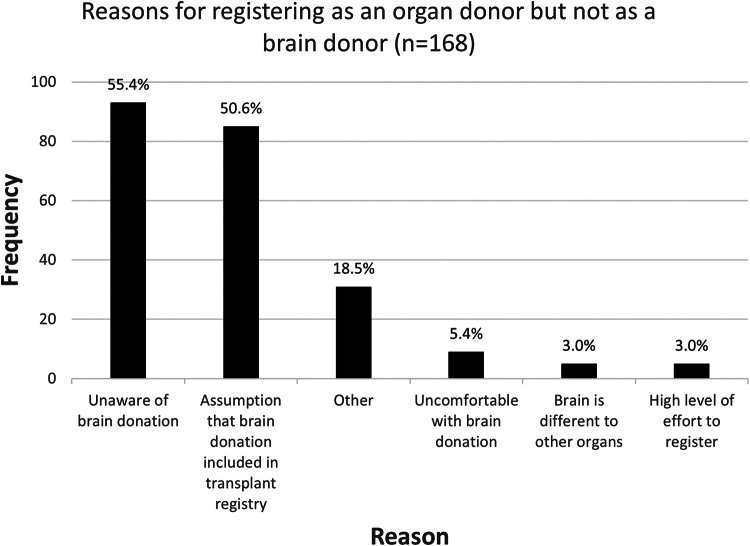
Reasons why organ donors were not brain donors. A histogram shows the ranked reasons why survey respondents who are registered as an organ donor for transplant have not yet enrolled as a brain donor for medical research. Respondents could choose up to five responses (unaware of brain donation, assume that brain donation is included in transplant registration, uncomfortable with brain donation, that they feel that the brain is different from other organs or it was a lot of effort to register) or provide a free text response under “Other.” Percentages are shown at the *top* of each bar.

Those respondents who had registered as organ or whole-body donors for transplantation or education (*n* = 170, 75%) were asked to select reasons why they had not registered for brain donation. Half of the respondents reported that they were not aware that registering for brain donation was possible (*n* = 93/170, 55%), and less than half of the respondents (*n* = 85/170, 50%), alternatively or also, reported that they assumed that brain donation was included when registering for organ donation for transplantation (Fig. 3).

**FIG. 4.**
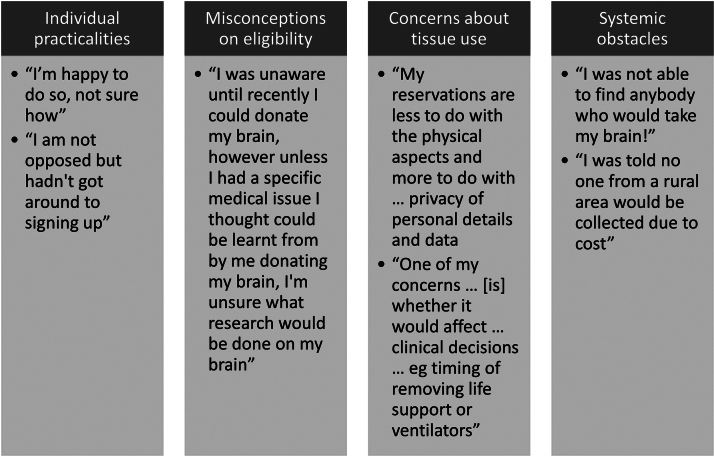
Free text responses about not being a brain donor. A table shows four themes as to why respondents had not yet registered with a brain donor program coded into themes (*dark gray boxes*, *top*). The quotes are in *light gray boxes*, *bottom*. Three researchers performed a thematic analysis to code each free text response as one of four themes.

Organ donors were also invited to report on their own reasons for not registering for brain donation, beyond those provided in the survey framework. Themes centered on individual practicalities (yet to commit to a decision, yet to register, or do not know how to register); misconceptions on eligibility (no need to study normal brains and being too young); concerns about use of tissue (privacy and effect on clinical decision-making); and systemic obstacles (no brain banks in the local area) (Fig. 4).

**FIG. 5.**
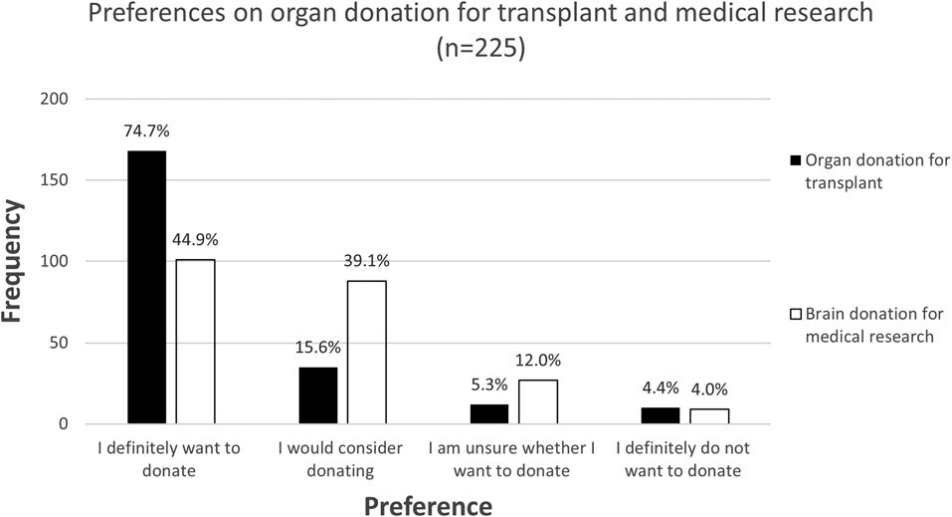
Support for organ and brain donation. A histogram shows the proportion of online survey respondents (*n* = 225) who would donate, consider donating, or were not willing to or undecided on donating their organs for transplantation (*shaded*) or their brain for future medical research (open). Respondents were able to select as many reasons as applicable from the provided list for signing up for any form of organ donation or choose “Other” and include an additional reason as free text (data not reported here). Percentages are shown at the *top* of each bar.

When all respondents were then asked about their planned actions on registering for brain donation, *n* = 101/225 (45%) indicated that they definitely wanted to donate their brain for medical research, with a further 88 (39%) reporting that they would consider brain donation (Fig. 5). The responses to this question did not differ according to reported income or rurality (*p* > 0.05; chi-square tests).

Six respondents (3%) stated that they had tried unsuccessfully to sign up for brain donation, while an additional three had tried unsuccessfully to sign up for whole-body donation. Only 9 respondents (4%) would definitely not donate their brain, with another 27 (12%) remaining unsure.

The top reason for opposing or being noncommittal on brain donation was that respondents did not like the thought of donating organs after death (*n* = 14/36, 39%), followed by a desire for their body to remain intact after death (*n* = 5/36, 14%). “Other” reasons included mistrusting the system, not getting around to it, and opposition from family members.

## Discussion

The aim of this study was to determine perceptions of brain donation among the general community in Australia. Due to the known high level of support for organ donation for transplant in Australia, we compared and contrasted attitudes toward brain donation in those who self-identified as organ donors versus those who did not. Our findings suggest that there is no aversion to the concept of brain donation for medical research, but that there are varying reasons for low donation rates. Indeed, only 4% of all respondents were opposed to brain donation.

A major preconception established in this study was that over half of the self-reported organ donors believed that brain donation for research was inclusive to their signing up as an organ donor for transplantation. For many organs, donation for either transplant or research is possible. However, for brains, there is no possibility of transplant and so provision of tissue for research, including consent, is not aligned with transplantation services and must be operated and marketed as a stand-alone operation.

Additional barriers to brain donation were identified when respondents were invited to report on their own barriers to brain donation, with predominant themes emerging on access to information on brain donation as well as ease of registration as a donor. Additional reports that highlighted misconceptions relating to donor characteristics, such as being too young, too sick, or too healthy, were consistent with previous research.^5,16^

The fact that many respondents assumed they were already registered as brain donors because of their organ donor status does suggest a general willingness for brain donation. However, it also indicates a widespread lack of understanding regarding exactly what registering as an organ donor through the Australian Organ Donor Register (AODR) entails.

This ambiguity is not exclusive to brain donation as a recent survey of 2056 students at The University of Sydney on whole-body donation revealed that many participants reported they had registered to donate their body to medical research through the AODR.^2^ Yet, this is not possible as the AODR program addresses organ donation for transplantation only.

The ubiquity of organ donation for transplantation in Australia is such that it is often referred to simply as *organ donation*. This terminology obscures forms of donation that fall outside the scope of transplantation, rendering them either invisible to the lay person or ambiguously conflated under this umbrella term.

It allows people to ignore the specific or pragmatic considerations of organ donation while engaging in altruism, although to do so, they must make several assumptions. Some comments by respondents indicated that not scrutinizing organ donation too closely was an intentional tactic that allowed them to avoid dwelling on their own mortality:

“I didn't think about the brain specifically when I signed up to be an organ donor… I think having to think of a specific organ for donation is more uncomfortable than the more general ‘organ’ donation, and also how ‘integral’ the brain is, one has to confront one's own mortality quite directly to consider the idea of the brain being used.”

“I have not really thought about specifically donating my brain. I have thought about donating my body but I admit I have done nothing about it. We all like to think we're not going to die!”

This is consistent with previous research, which found that social norms around avoidance of end-of-life discussions hinder the ability to even consider brain donation.^5,17,18^ Discussions with family are consistently reported as one of the most important factors when making organ donation decisions.^19–22^ Research has shown that having information available before death aids in decision-making for family members considering donating a loved one's brain.^5,8,23^

In the current study, approximately two-thirds of respondents had discussed organ donation with a family member, compared with less than one-fifth for brain donation. While the most common reason in our survey for not being a brain donor was a lack of awareness, with only seven respondents reporting having seen advertisements for it, one study found that people who had already heard of brain donation were 4.6 times more likely to register when approached.^24^ It is therefore important to normalize conversations regarding death and end-of-life decisions, including the potential opportunity for brain donation for research.

Notwithstanding a general avoidance of end-of-life discussions, previous studies have found that understanding how the brain is used for research has a positive influence on registering as a brain donor.^5,17,23,25^ Increasing awareness of how brain tissue is used and particularly the scientific advances resulting from its use could also reduce misconceptions that act as barriers to brain donation, as was acknowledged by one participant:

“I think it's important to provide specific information about brain donation science. Specifics matter with brains. I don't think anyone would care if their appendix ended up in a lab ferret, but they might have issues with their brain being transplanted whole into a sheep. If it's just bits, and cells etc., it's a whole lot less creepy. Be good to know before you found yourself part-sheep. (It's just an example. I know it wouldn't fit!)”

An unexpected barrier that was reported by respondents related to a lack of access to brain donation, with 3% of respondents stating that they had been unable to sign up for brain donation. It is unclear if this was because they did not fit criteria for the bank they had contacted, which may be disease specific or geographical, with banks often limiting their catchment areas to limit costs and postmortem delay.

### Limitations

The survey was conducted with a small sample size relative to the population of Australia. While some characteristics of the respondents reflected those nationally, most of the survey participants were women. A larger study with a more even mix of genders may reflect differing perceptions on brain donation. There was also little representation from the Aboriginal and Torres Strait Islander and culturally linguistically diverse (CALD) communities here. However, the representation of these groups reflects the current Australian population.^15^

### Future directions

The prominent levels of willingness and low levels of awareness of brain donation reported in this study are consistent with several studies globally.^5,17,18^ While increasing brain donation rates by removing barriers is a focus of much brain donation literature,^17,19–21^ it may be more ethical and efficient to simply promote brain donation as an available form of postmortem organ donation to individuals who would be willing, but unaware of the possibility.

Based on the results of this survey, increasing public awareness of all forms of organ donation is a crucial step toward increasing rates of brain donation. Increasing conversations about the specifics of brain donation will serve to demystify the organ donation processes and correct any misconceptions. This was a survey of the general community, but ultimately brain banks will need patients and health professionals who work directly with patients experiencing brain disorders to also serve as advocates for brain donation.

A follow-up survey looking at the barriers faced by clinicians in approaching and educating patients on brain donation would be beneficial.

## Conclusions

This study reinforces the prominent public support for brain donation in Australia, with an encouraging 84% of people willing to donate their brain in the future. Most participants in this survey expressed a desire to donate their organs postmortem in whatever way is most useful, but had little knowledge of how to do so. They also held various misconceptions about the registration process and how their tissue would be used.

Rather than convincing unwilling individuals of the importance of brain donation, improving access and increasing education emerged as key factors in enabling already willing populations to sign up for brain donation. The prevalence of misconceptions in public understanding of organ donation, including conflation of organ donation for transplantation and brain donation for research, indicates the need for widespread education on the specifics of all types of postmortem organ donation so that individuals may make informed decisions.

For those respondents who were already well informed on organ donation for medical research, access was the primary barrier to brain donation. This indicates that low brain donation rates are not solely an issue with awareness but that broader structural shortcomings are also a contributing factor.
