# Supplementary material for: A Survey of Community Perceptions on Brain Donation for Research
Source: Biopreserv Biobank. 2025 Feb 13;23(1):31–8. doi: 10.1089/bio.2023.0158 (PMC12344117; doi:10.1089/bio.2023.0158)
Supplement: Supplementary Data S1 [file bio.2023.0158_suppl_datas1.docx]

Supplementary data

**Survey**

In Australia, post-mortem organ donation is undertaken for three distinct purposes: transplantation, teaching purposes, or for medical research into specific diseases. These all have different registration processes, with organ donation for transplantation the most well-known and widely practiced. Unlike organ donation for transplant which has one all-encompassing national register, organ donation for medical research is facilitated by various biobanks across Australia. The NSW Brain Tissue Resource Centre at the University of Sydney is a brain bank that primarily focuses on facilitating research into the causes and development of alcohol use disorders, mental health disorders and ageing. Our mission is to enable advances in medical research into neurological illnesses to understand, treat and cure disease.

| **Question** | **Question type/allowable responses** |
| --- | --- |
| *Part 1* | |
| 1. Age | [free text] |
| 1. Gender | Drop down options:   - Female - Male - Non-binary - Prefer not to respond |
| 1. Marital status | Drop down options:   - Single - Married - De facto - Divorced - Widowed - Separated - Prefer not to respond |
| 1. Is English the main language spoken in your home? | [Yes/no] |
| 1. With which racial or ethnic group(s) do you most identify? | Drop down options:   - Caucasian - Aboriginal, Torres Strait Islander or Pacific Islander - African - Asian - Middle Eastern - Hispanic - Other |
| 1. What are your religious beliefs? | Drop down options:   - Christian - Muslim - Buddhist - Agnostic - No religion - Other (please specify) |
| 1. What is your highest level of education? | Drop down options:   - Less than Year 12 - Year 12 or equivalent - TAFE/vocational qualification/trade certificate - Undergraduate degree - Postgraduate degree - Doctoral degree |
| 1. What is your personal income level per annum? | Drop down options (AUD):   - $0 – 18200 - $18201 – 37000 - $37001 – 90000 - $90001 – 180000 - Prefer not to respond |
| 1. On what basis are you employed? | Drop down options:   - Casual - Contract - Full time - Part time - Unemployed - Student - Self-funded retiree - Age pensioner - Other (please specify) |
| 1. Postcode | [free text] |
| 1. Which of the following best describes where you live? | Drop down options:   - Urban - Rural - Remote |
| *Part 2* | |
| 1. Do you experience any of the following? | Drop down options (select all that apply):   - Alcohol misuse - Mental illness - Alzheimer’s disease/Dementia - Other brain disease/disorder (please specify) |
| 1. Have you ever considered either/both organ donation for transplantation or medical research? | Yes/no |
| 1. How comfortable are you talking about organ donation? | Likert Scale:   - Very comfortable - Moderately comfortable - Neither comfortable nor uncomfortable - Moderately uncomfortable - Very uncomfortable |
| 1. Select all that apply: | Drop down options:   - I am a registered organ donor for transplant - I am a whole body donor for medical education - I am an active participant of a brain donation program for medical research - I am none of the above - I am unsure |
| 1. If you are an organ donor for transplant, how did you register? | Drop down options:   - Online through DonateLife - Indicated on driver's license - Other (please specify) - N/A - Unsure |
| 1. If you are a brain or organ donor, what were your reasons for signing up? | Drop down options (select all that apply):   - After I die my organs will be of no use to me - Seems like the right thing to do/altruism/helping others - To advance medical research - I think after I die my family will find comfort in the idea that my death was contributing to society - The importance of organ donation was brought home to me when a friend/family member experienced disease - Other - N/A |
| 1. If you are an organ donor for transplant and are not registered with a brain donation program, what is the reason for this? | Drop down options (select all that apply):   - I did not know you could donate your brain - I thought brain donation was included when I registered as an organ donor - I am uncomfortable with brain donation - It seems like a lot of effort - I believe the brain is different to other organs - I am not a brain or organ donor - I am both a brain and organ donor - Other (please specify) |
| 1. If you are neither a brain or organ donor and you are unwilling to sign up, what are your reasons? | Drop down options (select all that apply):   - Religious beliefs - I don’t like the thought of it - I believe the body should be kept intact after death - I don’t believe I am healthy enough - Other (please specify) - N/A |
| 1. Regardless of your personal donor status, what do you think of organ donation for transplantation in principle? | Likert Scale:   - Strongly support the principle of organ donation for transplantation - Support the principle of organ donation for transplantation - Neither support nor oppose the principle of organ donation for transplantation - Oppose the principle of organ donation for transplantation - Strongly oppose the principle of organ donation for transplantation |
| 1. Regarding your personal decisions toward organ donation for transplantation, which of the following statements is most accurate: | Drop down options:   - I definitely want to donate all my organs - I would consider donating all my organs - I definitely want to donate some of my organs - I would consider donating some of my organs - I am unsure whether I want to donate any organs or not - I definitely do not want to donate any organs |
| 1. Regarding organ donation for transplant, how confident are you that your family would carry out your wishes after your death if you wished to donate? | Likert Scale:   - Very confident - Confident - Fairly confident - Not very confident - I’d rather not say |
| 1. Regardless of your personal donor status, what do you think of brain donation for medical research in principle? | Likert Scale:   - Strongly support the principle of brain donation for medical research - Support the principle of brain donation for medical research - Neither support nor oppose the principle of brain donation for medical research - Oppose the principle of brain donation for medical research - Strongly oppose the principle of brain donation for medical research |
| 1. Regarding your personal decisions toward brain donation for medical research, which of the following statements is most accurate: | Drop down options:   - I definitely want to donate my brain for medical research - I would consider donating my brain for medical research - I am unsure whether I want to donate my brain or not - I definitely do not want to donate my brain |
| 1. Regarding brain donation for medical research, how confident are you that your family would carry out your wishes after your death if you wished to donate? | Likert Scale:   - Very confident - Confident - Fairly confident - Not very confident - I’d rather not say |
| 1. Have you discussed organ donation for transplant with: | Drop down options (select all that apply):   - Doctor - Family - Friends - Next of Kin - Carer - Health researchers - No-one - Unsure - Other (please specify) |
| 1. Have you discussed brain donation for medical research with: | Drop down options (select all that apply):   - Doctor - Family - Friends - Next of Kin - Carer - Health researchers - No-one - Unsure - Other (please specify) |
| 1. Have you seen advertisements for organ donation for transplantation? | Yes/no |
| 1. Have you seen advertisements for brain donation for medical research? | Yes/no |
| 1. If you have any additional comments please enter them here | [free text] |
